# TAVI for Bicuspid Aortic Valve: Addressing Technical Challenges and Optimizing Outcomes

**DOI:** 10.3390/jcm14217860

**Published:** 2025-11-05

**Authors:** Donato Antonio Paglianiti, Cristina Aurigemma, Marco Busco, Luigi Cappannoli, Francesco Bianchini, Enrico Romagnoli, Mattia Lunardi, Francesco Fracassi, Lazzaro Paraggio, Antonino Buffon, Rocco Antonio Montone, Antonio Maria Leone, Carlo Trani, Francesco Burzotta

**Affiliations:** 1 Department of Cardiovascular and Pulmonary Sciences, Università Cattolica del Sacro Cuore, 00168 Rome, Italy; 2 Department of Cardiovascular Sciences CUORE, Fondazione Policlinico Universitario A. Gemelli IRCCS, 00168 Rome, Italy; 3 Centro di Eccellenza in Scienze Cardiovascolari, Ospedale Isola Tiberina Gemelli Isola, 00186 Rome, Italy

**Keywords:** bicuspid aortic valve, transcatheter aortic valve replacement, aortic stenosis, pre-procedural Imaging

## Abstract

Bicuspid aortic valve (BAV) is the most common congenital valvular anomaly, affecting roughly 1–2% of the population and predisposing to premature aortic stenosis and thoracic aortopathy. Surgical aortic valve replacement (SAVR) remains the standard therapy, while transcatheter aortic valve implantation (TAVI) is increasingly adopted across a broader range of risk profiles due to accumulating evidence and advancements in device technology. Observational registries and early trial data indicate that TAVI is technically feasible in selected BAV anatomies, with device-success rates exceeding 90%. Nonetheless, bicuspid morphology is still technically demanding, with several possible pitfalls during transcatheter procedure and pre-procedural planning compared with tricuspid valve. The rates of moderate-to-severe paravalvular leak (PVL), permanent pacemaker implantation (PPI), and annular complications remain high, especially in the presence of extensive calcifications with raphe or tapered roots, underscoring the importance of meticulous multimodality imaging, dedicated sizing algorithms, and device-specific strategies. Long-term durability signals are encouraging but remain limited, underlining the need for prospective trials with extended follow-up. This review summarizes current knowledge on BAV anatomy and its management, exploring the available evidence supporting the role of transcatheter approach in this challenging and unique scenario.

## 1. Introduction

Bicuspid aortic valve (BAV) is a common congenital malformation (1–2% prevalence in the general population) often associated with other congenital defects and aortopathy. This broad and heterogeneous spectrum ranges from complex syndromic presentations to isolated valvular dysfunction, typically leading to early-onset stenosis/regurgitation and thoracic aorta dilatation. Because of these anatomical complexities and the frequent association with aortic pathology, BAV-related aortic stenosis has traditionally been managed surgically. Transcatheter aortic valve implantation (TAVI) procedure is a well-established treatment for aortic stenosis (AS) in high and intermediate surgical risk patients [1,2], with emerging and promising results also in younger and low risk scenarios [3,4]. Randomized controlled trials (RCTs) supporting the widespread adoption of TAVI in clinical practice have typically excluded patients with bicuspid anatomy. Nevertheless, real-world data show that approximately 10% of elderly patients undergoing TAVI present with a BAV, reflecting the growing need to better define the role of the transcatheter approach in this context. Given the unique anatomical and clinical challenges posed by BAV and the limited dedicated evidence currently available, the optimal management strategy remains a subject of ongoing discussion.

This review aims to provide an updated synthesis of anatomical classifications, technical considerations, procedural strategies, and clinical outcomes of TAVI in BAV patients (graphical abstract).

## 2. Anatomical Classification

Bicuspid aortic valve disorders encompass a large spectrum of anatomical and clinical conditions for whom appropriate management and prognosis may differ significantly. Recent real-world data show a marked prevalence of BAV (49.5%) among male patients younger than 65 years undergoing SAVR, with progressive reduction in the elderly population (19.1% between 65 and 79 years, and only 4.5% in octogenarians) [5].

**Figure 1:**
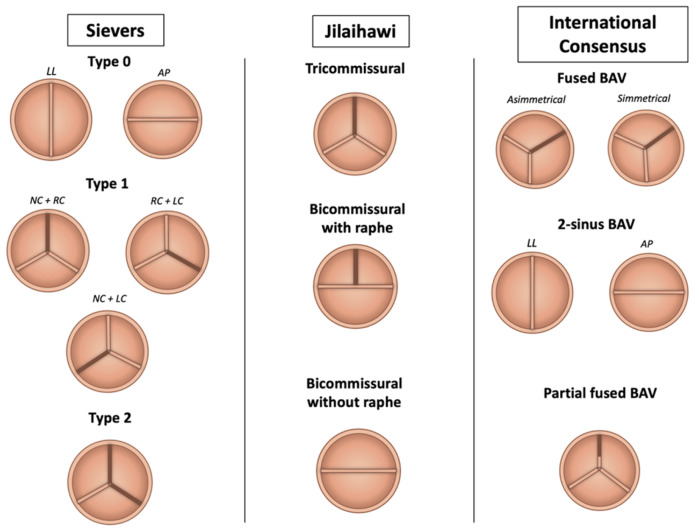
Anatomical classification of bicuspid aortic valve phenotypes. Abbreviations: AP = antero-posterior; BAV = bicuspid aortic valve; LC = left cusp; LL = latero-lateral; NC = non-coronary cusp; RC = right-cusp.

Typically, BAV is a congenital valve abnormality characterized by the presence of one/two anatomical or functional aortic valve leaflets instead of three, but different grades of valve-apparatus distortion, along with valve function, may occur. For this reason, a huge effort over the years has been made to try to classify the morphological phenotypes of BAV disease (Figure 1).

The Sievers and Schmidtke system is the most widely used, identifying type 0 (two symmetric leaflets without raphe), type 1 (one raphe, with subtypes based on raphe location), and type 2 (two raphes forming a tricuspid-like configuration) [6]. In the context of TAVI, Jilaihawi et al. proposed a CT-based classification focusing on commissural anatomy: bicommissural with raphe (similar to Sievers type 1), bicommissural without raphe (type 0), and tricommissural valves, which more closely resemble tricuspid anatomy [7]. The proposed classification seeks to address the specific challenges of TAVI in the management of BAV stenosis, focusing on how the presence and characteristics of the valve apparatus may influence the performance of implanted prosthesis. The presence of the raphe, especially if congenital compared with the acquired one, limits the appropriate expansion of prosthesis, increasing the risk of paravalvular leaks (PVLs) and the technical failure of TAVI [7] The Michelena consensus added a broader framework, categorizing BAV into three morphologies: fused BAV (90–95% of cases), characterized by raphe between fused cusps; 2-sinus BAV, with two symmetric cusps and sinuses; and partial-fusion BAV, featuring incomplete cusp fusion. These anatomical variants, along with features like calcification distribution and raphe characteristics, are critical to pre-procedural planning and the decision between TAVI and surgical approaches [8]. The most common BAV presentation is the “typical valvulo-aortopathy condition”, with progressive BAV dysfunction and/or aorta dilatation without major associated or concomitant disorders. Three different types of BAV anatomies were identified: the “fused BAV” (accounting for 90–95% of the cases [8,9]), which is characterized by the fusion of two aortic cusps by a congenital fibrous ridge (the raphe, located in 70–80% between right and left cusps, 20–30% right and non-coronary cusps, 3–6% left and non-coronary cusps) and three distinct aortic sinus; the “2-sinus BAV” morphology (5–7% of the cases [9]), with two symmetrical aortic sinus and two different cusps (latero-lateral or anterior-posterior phenotype according to the anatomical pattern); the “partial-fusion BAV” morphology (whose prevalence is unknown) with three different sinus and cusps and a small ‘mini-raphe’ (less than 50% of cusps fusion), described mostly in the operating room in patients undergoing surgery for aorta dilatation [10].

The presence and distribution of calcifications of raphe and leaflets deeply influence the outcomes of the procedure [11]. Thus, the phenotype of the valve apparatus and the “high-risk” features of specific subgroups should guide the pre-procedural planning and the appropriate decision between TAVI or surgery. Several features have been identified as high-risk or technically challenging for TAVI. These include heavily and asymmetrically calcified raphes, excessive leaflet or annular calcification, extremely large annular dimensions, shallow or effaced sinuses of Valsalva, extensive LVOT calcification, marked aortopathy (particularly ascending aorta dilation >45 mm), and excessive angulation between the aortic annulus and the ascending aorta. In such scenarios, surgical aortic valve replacement (SAVR) may be favored due to the greater control it allows in addressing complex anatomy and associated aortic pathology.

Summary: Over time, various classification systems have been proposed to address the heterogeneity of BAV disease in both research and clinical settings. While the Sievers system remains the most used, the Jilaihawi classification is particularly useful for evaluating calcification patterns and anticipating TAVI-related challenges. The most recent consensus classification is more comprehensive for clinical and research purposes but has limited utility in pre-procedural transcatheter planning.

## 3. Current Guidelines and the Unmet Need in Bicuspid Aortic Valve Stenosis Management

Despite significant advancements in TAVI procedures and the progressive shift from high-risk to intermediate and low-risk scenarios, the current guidelines still reflect limited evidence in the management of BAV stenosis. The general indications for treatment of severe aortic valve stenosis without associated aortopathy do not differ between tricuspid or bicuspid anatomy and are based on symptoms and cardiac function. However, some discrepancies exist regarding the choice of valvular intervention. The previous 2021 ESC/EACTS guidelines of valve disease [1] report no specific recommendations regarding the transcatheter approach for bicuspid one. According to the new 2025 ESC/EACTS guidelines [12], SAVR remains the cornerstone for the treatment of stenotic BAV and associated conditions, with TAVI considered only in patients deemed ineligible for surgery, if anatomy is suitable (class IIb of recommendation). Similarly, the 2020 ACC/AHA guidelines [2] are also conservative regarding BAV-stenosis management: TAVI is considered as an alternative to surgery in patients with BAV stenosis at increased surgical risk, provided that an individualized assessment confirms favorable anatomy, procedural feasibility, and acceptable risk profile.

Summary: Current guidelines limit the TAVI to patients at high surgical risk. However, if evidence grows and new data are available, the guidelines may adapt, potentially broadening the scope of TAVI in the BAV patient population.

## 4. Technical Challenges

TAVI performed on BAV stenosis presents several possible pitfalls and challenges that distinguish it from TAVI in tricuspid aortic valve stenosis. Morphological features of bicuspid anatomy, such as extensive leaflets calcification and the presence of a calcified raphe, may significantly influence TAVI outcomes. Yoon et al. found that patients with both of these characteristics, treated with TAVI, experienced a higher risk of procedural complications with a fourfold increase in mortality at 2 years [11]. Moreover, a recent study by Li et al. [13], conducted on a multinational retrospective cohort of 2553 patients undergoing TAVI, showed that BAV-0 patients were associated with a better long-term prognosis compared with TAV and BAV-1. This suggests that the absence of a raphe and the presence of a symmetrical morphology allows more uniform prosthesis expansion rather than the asymmetrical displacement seen in raphe-dominant anatomies, potentially leading to improved clinical outcomes. This benefit was maintained even when compared with the TAV subgroup, demonstrating how patients with BAV are heterogeneous, and highlighting the relevant impact of the anatomical features on procedural and clinical results.

Abnormal aortic valve morphology and concomitant ascending aortic tract dilatation lead to different technical challenges during the TAVI procedure that range from pre-procedural planning to THV deployment. Difficulty in finding an adequate implant projection, challenging crossing, and advancement of the delivery system across the aortic valve or marked instability of the THVs during deployment (particularly of SE-THV in tapered anatomies) are possible pitfalls that can increase the risks of sub-optimal procedure results. In this context, the knowledge of peculiar tips, such as the use of stiffer guidewires, snaring devices, delivery systems with active flexible or adequate pre-dilatations, may help interventional cardiologists to overcome these challenges [14].

### 4.1. THV Sizing and Implantation Depth

Typically, the sizing of the aortic prosthesis is based on the dimensions of the virtual basal ring (VBR), the tightest part of the aortic root, identified through multislice computed tomography (MSCT) imaging by merging the hinge points at the lowest insertion of the three aortic cusps. Bicuspid anatomy is often characterized by asymmetric cusps (especially for non-fused ones in the type-1 BAV scenario), extensive eccentric calcifications, and elliptical shape of the annulus. These features make the identification of a clear VBR particularly challenging, and consequently the choice of an appropriate size and positioning of THVs during pre-procedural planning.

**Figure 2:**
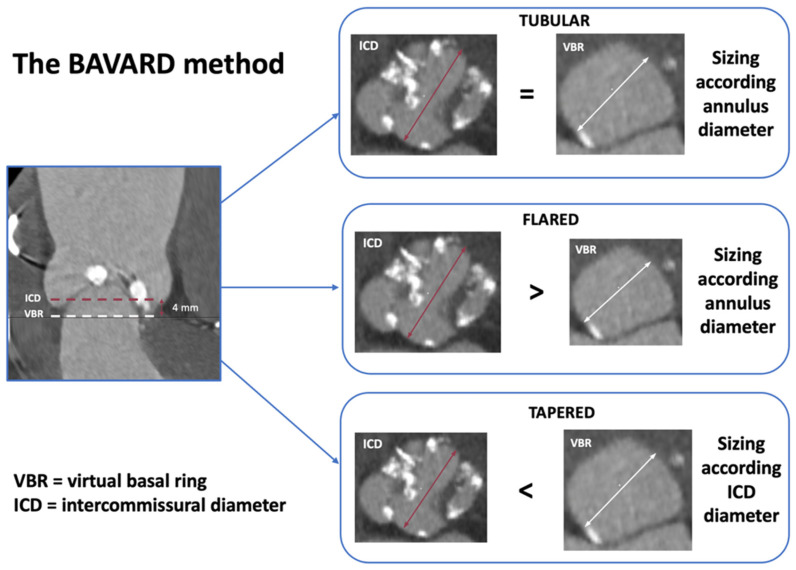
BAVARD algorithm for annular sizing in bicuspid aortic valve. Abbreviations: BAVARD = Bicuspid Aortic Valve Anatomy and Relationship with Devices; ICD = intercommissural diameter (red arrow/dash-line); VBR = virtual basal ring (white arrow/dash-line).

**Figure 3:**
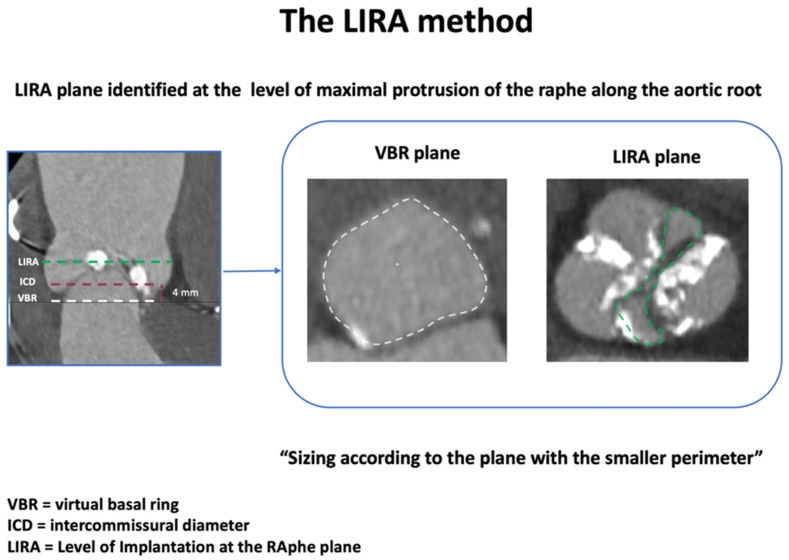
LIRA method for pre-procedural sizing in bicuspid aortic valve. Abbreviations: LIRA = Level of Implantation at the Raphe (green dash-line); ICD = intercommissural diameter (red dash-line); VBR = virtual basal ring (white dash-line).

**Figure 4:**
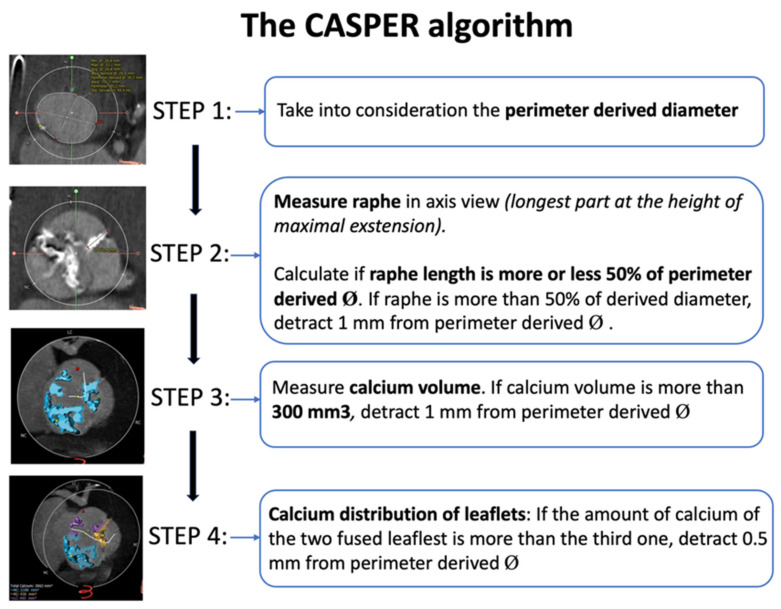
The CASPER algorithm for pre-procedural sizing in bicuspid aortic valve. Abbreviations: CASPER = Calcium Algorithm Sizing for bicusPid Evaluation with Raphe.

**Figure 5:**
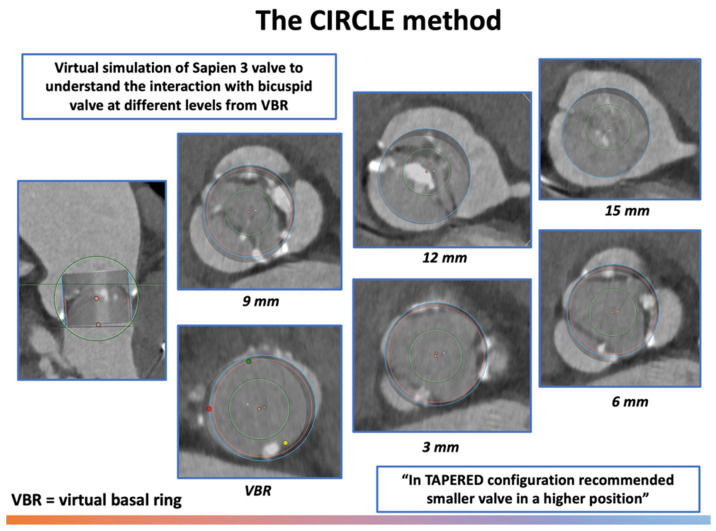
The CIRCLE method for pre-procedural sizing in bicuspid aortic valve. Abbreviations: VBR = virtual basal ring.

To overcome this variability and predict the behavior of implanted prosthesis with the native valve apparatus, different THV sizing methods have been proposed over the years (Table 1, Figure 2, Figure 3, Figure 4 and Figure 5).

The BAVARD (Bicuspid Aortic Valve Anatomy and Relationship With Devices) registry [15], based on 101 TAVI treated BAV-patients, mainly type 0 or 1 by Sievers et al. [6], classified the BAV morphologies in three different configurations according to the annular diameter (measured at standard VBR) and inter-commissural distance (ICD, measured at 4 mm above the annulus): “tubular” (annulus diameter = ICD), “flared” (annulus diameter < ICD), and “tapered” (annulus diameter > ICD) ones (Figure 2).

This classification aims to highlight the potential points of constraints throughout the aortic root that can lead to THV underexpansion and hamper prosthesis durability or promote leaflet thrombosis. In most cases (tubular and flared anatomies, 88% of patients), THV sizing should be performed on true annulus diameter, only with minimal oversizing (3–4%). Conversely, in “tapered” configurations, the level of ICD (measured at 4 mm above annulus) represents the main determinant of prosthesis constriction. Relying solely on annular-based sizing in these cases could lead to the selection of a device that is potentially too large. Therefore, selecting a smaller THV size based on ICD and on the distribution of calcium and raphe is generally preferred. However, in the prospective BIVOLUTX registry [16], similar procedural and clinical outcomes were observed when comparing annular-based sizing and combined sizing (annular plus supra-annular measurements) with self-expanding THVs.

In the large-scale retrospective “AD-HOC registry”, enrolling consecutive stenotic Sievers type 1 BAV treated with TAVI, similar results were obtained when comparing, after propensity score matching, annular versus supra-annular sizing strategies in the tapered population [17], yielding comparable rates of technical success, 30-day device success, and early safety outcomes. Nonetheless, a slightly higher post-TAVI gradient (mean +2 mmHg) was observed in the supra-annular group.

Another BAV-specific sizing strategy is the Level of Implantation at the RAphe (LIRA) method, as originally described by Iannopollo et al. [18] in patients with raphe-type BAV disease. Briefly, this supra-annular sizing method combines the measurement of baseline CT scan perimeters at the LIRA plane, defined as the level of maximal protrusion of the raphe along the aortic root, and at the virtual basal ring. The perimeter at the LIRA plane is obtained by tracing the internal border of the leaflets, excluding any calcific or non-valvular structures encountered at this level (Figure 3).

In the case of a discrepancy of measurements between the two planes, the prosthesis is sized according to the plane with the smaller perimeter, leading to the selection of smaller-sized prostheses. The LIRA sizing approach is based on a bidimensional parameter (the perimeter) rather than the ICD (unidimensional measure) proposed in the above-mentioned BAVARD algorithm. Furthermore, the LIRA plane is not located at a fixed distance above the VBR but adapts to the anatomical variability. The feasibility of this strategy has been initially validated in a small cohort of twenty prospective raphe-type BAV patients, showing favorable procedural outcomes: 100% VARC-2 device success, low trans-prosthetic gradients, and no significant paravalvular leak (PVL) [18].

The Calcium Algorithm Sizing for bicusPid Evaluation with Raphe (CASPER) method is another strategy proposed for THV sizing in 2019 [19]. The CAPSER algorithm integrates the measurement of diameter/area at the level of VBR with three other parameters that significantly influence prosthesis expansion: raphe level/length, maximum calcium burden, and its distribution. First, VBR measurements must be calculated, and in the case of high calcium score (>300 mm^3^) and extensive raphe length (more than 50% of area/perimeter derived mean diameter at the annulus level), 1 mm should be subtracted from the initial annulus measurements for TAVI undersizing. Furthermore, if the calcium burden is predominantly located mainly at the raphe-site, an additional correction of 0.5 mm should be considered for final sizing (Figure 4).

This algorithm was validated in a cohort of 21 type I BAV patients, showing favorable procedural outcomes with successful TAVI performance [19].

A dedicated sizing and positioning approach was proposed in 2021 for balloon expandable valve (BEV) (Sapien 3/Ultra, Edwards Lifesciences) in bicuspid anatomy [20]. The Circle method for supra-annular sizing involves projecting a virtual circle on MSCT images of the identical diameter to the proposed SAPIEN valve at different distances from the annulus (0–3–6–9 mm) to assess the sealing zone and determine both the valve size and optimal implantation depth (Figure 5).

This strategy provides useful information regarding the interaction between the implanted THV and the bicuspid anatomy, further suggesting the optimal implantation level. In most cases, the traditional implantation depth ranges between 80/20 and 90/10 (aortic/ventricular stent frame height), however, for complex anatomies where supra-annular rather than annular sealing and anchoring is needed (tapered configuration), a higher implantation depth is suggested (90/10 or 100/0).

Balloon pre-dilatation can be used as an additional tool for sizing. Gentle pre-dilation with a semi-compliant balloon according to the smaller annular diameter, combined with simultaneous aortography, may provide valuable insights into annular and supra-annular distensibility, the risk of paravalvular leak (PVL) or coronary obstruction, and may help identify the level of the balloon waist (indentation) above the annulus. This sign may guide operators in considering the level of indentation as a primary determinant of valve anchoring or constriction, and in selecting the prosthesis accordingly. However, this method should be integrated with comprehensive imaging-based analysis, especially in “grey-zone” cases.

Computed tomography is essential to guide transcatheter heart valve (THV) sizing in bicuspid aortic valve (BAV) patients. Type-0 BAV features two symmetric cusps without a raphe, while Type-1 often presents with a heavily calcified raphe, which increases procedural risks. For raphe-type anatomies, we used the supra-annular “LIRA” sizing method, selecting the smaller perimeter between the annulus and the raphe level, which has shown excellent outcomes in our series.

Valve selection is anatomy-dependent (Table 2). Supra-annular self-expanding valves are preferred in large or tapered annuli due to superior effective orifice area (EOA) and lower gradients. Intra-annular balloon-expandable valves may be suited for more symmetric anatomies, but require careful sizing to avoid oversizing-related complications. In highly calcified or elliptical annuli, we favor a conservative “downsizing” approach to reduce the risk of annular rupture, even if this results in a slightly smaller EOA. Supra-annular valve design can partially offset this trade-off. Finally, bulky commissural calcification is a strong predictor of suboptimal results, warranting extra caution during planning.

### 4.2. THV Post-Dilatation

Balloon post-dilatation should be considered only in cases where significant PVL or elevated transvalvular gradients are detected. The risk of valve and calcium embolization, aortic injury, and conduction disturbances must be carefully considered and balanced when approaching post-implantation balloon optimization. In cases of large annuli beyond the range of currently available THVs, over-expanding and overfilling remains a possible alternative; using the “flare outflow” technique may allow for proper sealing and anchoring [21].

### 4.3. Tortuous or Horizontal Aortas

These anatomic variants significantly complicate delivery and alignment of the THV. A horizontally oriented aorta is a factor that can make TAVI in BAV cases more challenging. Operators typically use highly supportive guidewires and steerable introducer sheaths to navigate difficult arches. In some cases, a snare is deployed through the arterial catheter to centralize a self-expanding delivery system within the ascending aorta [14]. Balloon-expandable valves are often preferred in horizontal aortas because their delivery catheters are more flexible; conversely, self-expanding THVs may require the use of a snare or controlled flexion to avoid aortic injury.

### 4.4. Valve Crossing

Marked calcification or a calcified raphe can obstruct catheter passage, requiring additional maneuvers. Adequate predilation with a large balloon is essential to fracture calcium. Using delivery systems with more flexibility (e.g., balloon-expandable THVs) can improve trackability. Adjunctive techniques include the “buddy-balloon” maneuver (inflating a second balloon alongside the leaflets to align the prosthesis) and snaring a guidewire to pull the delivery catheter through tight calcific gaps [14]. These techniques help guide the THV tip safely across the stenotic orifice.

### 4.5. Very Large Annuli

Extremely wide annular diameters (beyond device sizing charts) pose a contraindication to standard THVs. Some BAV geometries have a “volcano” shape where the supra-annular space can be smaller than the annulus. In this case, ‘undersized’ THVs anchored supra-annularly at the raphe can still achieve a seal. In such anatomies, annular-based sizing alone may underestimate the potential for prosthesis sealing. Modestly undersized THVs can still achieve adequate anchoring and sealing when deployed at the level of the raphe or supra-annular waist, especially in tapered configurations. This technique leverages anatomical constraints at the supra-annular level for stabilization while reducing the risk of annular rupture associated with excessive oversizing. However, careful case-by-case evaluation is essential, as this strategy may come at the expense of increased paravalvular leak or suboptimal hemodynamics in less favorable geometries. Pre-procedural assessment with imaging and the consideration of alternative anchoring planes are critical when planning TAVI in patients with BAV and very large annuli.

Summary: Multiple strategies have been proposed for appropriate pre-procedure planning, each of these highlighting specific features and pitfalls of TAVI in BAV patients (Table 1, Figure 2, Figure 3, Figure 4 and Figure 5). Although most cases can be managed with the traditional sizing/implantation approach, there are specific anatomies (tubular ones) where careful imaging-based planning and algorithm-decision integration are essential to address the intrinsic complexity and optimize procedural outcomes.

### 4.6. Aortic Root and Ascending Aorta Dilatation in BAV Patients Undergoing TAVI

Anatomical characteristics can significantly impact procedural success, and consequently, clinical outcomes in bicuspid aortic valve (BAV) patients undergoing TAVI. Ascending aortic dilatation is frequently encountered due to inherent structural abnormalities of the aortic media [8]. Current ESC/EACTS guidelines recommend surgical replacement of the ascending aorta during SAVR when the diameter exceeds 45 mm in patients with BAV, particularly in the presence of additional risk factors such as rapid aortic growth (>3 mm/year) or a family history of aortic dissection [12]. In the context of TAVI, however, the management of associated aortopathy remains controversial. Recent observational studies have shown that post-TAVI aortic expansion tends to be modest in patients with untreated ascending aorta dilatation, especially when the baseline diameter is <50 mm [11]. Nonetheless, long-term surveillance with serial imaging is recommended, particularly in younger patients or those with borderline aortic dimensions. Currently, an ascending aorta diameter >50–55 mm is generally considered a relative contraindication to TAVI pending stronger prospective evidence.

**Figure 6:**
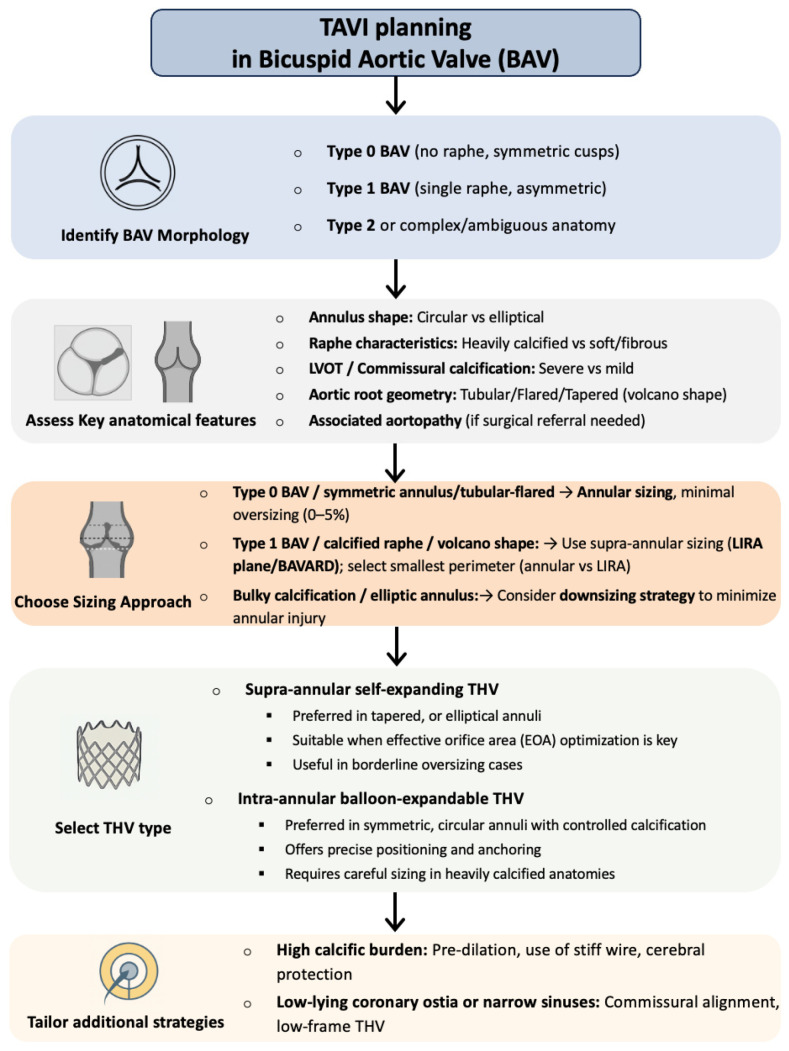
Algorithm for TAVI planning in bicuspid aortic valve (BAV) disease. Abbreviations: BAV, bicuspid aortic valve; BEV, balloon-expandable valve; EOA, effective orifice area; LIRA, Level of Implantation at the RAphe algorithm; LVOT, left ventricular outflow tract; SEV, self-expanding valve; TAVI, transcatheter aortic valve implantation; THV, transcatheter heart valve.

A summary algorithm integrating morphological classification, sizing strategy, and THV selection is presented in Figure 6.

## 5. TAVI Outcomes in Bicuspid Anatomy

Current evidence supporting TAVI among patients with BAV anatomy is scarce and limited to large-scale retrospective and prospective registries. Despite quite negative first experiences, contemporary data suggest the feasibility of the TAVI procedure with newer-generation THVs in favorable anatomies. A summary of the evidence described is reported in Table 3.

### 5.1. TAVI vs. SAVR

Direct comparisons between surgical and transcatheter management are based on observational registries, which generally favor SAVR in younger patients without comorbidities, particularly when concomitant aortopathy is present [34].

Elbadawi et al. [22] conducted a propensity score matching study comparing 975 BAV patients treated from 2012 to 2016 with TAVI versus SAVR, respectively. Data were retrospectively collected from codes of large healthcare databases. During the referred period, the percutaneous approach was increasingly utilized among bicuspid patients, with similar in-hospital mortality (3.1% vs. 3.1%, *p* > 0.999) between both groups. However, TAVI was associated with a higher incidence of conduction abnormalities (14.9% vs. 6.2%, *p* < 0.001) and subsequent permanent pacemaker insertion (13.8% vs. 4.6%, *p* < 0.001) compared with surgery.

Similarly, consistent evidence was provided by another retrospective propensity scores-based analysis conducted on 699 BAV patients undergoing TAVI versus SAVR [23]. At midterm follow-up (30 days and 1 year), no difference was observed in mortality (1-year: 9.0% vs. 7.4%), stroke (1-year: 4.0% vs. 3.7%) and HF hospitalization (1 year: 2.3% vs. 2.9%) with TAVI compared with SAVR. However, patients treated with TAVI had a higher incident of in-hospital new pacemaker implantation (12.2% vs. 7.6%). Furthermore, in another propensity score-matched cohort (1393 BAV patients treated in the USA between 2016 and 2018), TAVI was associated with reduced risk of in-hospital mortality (0.7% vs. 1.8%, *p* = 0.035) and a similar risk of 30-day (1% vs. 1.5%) and six-month MACE (4.2% vs. 4.9%) compared with SAVR [24].

The NOTION-2 (Nordic Aortic Valve Intervention) trial [25] is the only randomized study evaluating the impact of TAVI in low-risk patients (median STS 1.1%), including both bicuspid and tricuspid valve anatomies. Despite being underpowered for statistical inference, the subgroup analysis derived from 100 BAV patients (51 SAVR vs. 49 TAVI) highlighted a non-significant trend toward a higher incidence of the primary composite endpoint (death, stroke and rehospitalization) in TAVI compared with SAVR (14.3% vs. 3.9%; *p* = 0.07). Patients with BAV presented also more disabling strokes and moderate or severe PVL compared with SAVR.

Summary: Taken together, although randomized data are lacking, the available evidence suggests that TAVI may represent a feasible treatment option in selected patients with favorable bicuspid anatomy and no significant aortopathy. However, these findings should be interpreted cautiously, and further data, particularly from prospective randomized trials, are needed to confirm the safety and efficacy of TAVI in low-risk bicuspid populations.

### 5.2. TAVI in BAV vs. TAVI in TAV

Subgroup analysis of BAV patients managed with TAVI in the above-mentioned NOTION-2 trial [25], provides additional evidence when comparing tricuspid versus bicuspid anatomy. In the latter group, the risk of significant (moderate or severe) PVL was threefold higher compared with the tricuspid cohort (9.1% vs. 3.1%), without enhancing the rate of new permanent pacemaker implantation (14.6% vs. 15.2%). A trend of more composite of death, stroke, and rehospitalization was also noticed (8.7% vs. 14.3%). Again, considering the indirect nature of comparisons performed and the lack of statistical power as a subgroup analysis, these data should be interpreted with caution.

A study by Makkar et al. [26] comparing TAVI outcomes in low-surgical risk patients with bicuspid and tricuspid anatomy (3168 propensity-score matched pairs between two cohorts from STS/ACC TVT Registry, treated with balloon-expandable Sapien3 transcatheter heart valve) showed no significant differences in mortality at 30 days (2.6% vs. 2.5%) or 1 year (10.5% vs. 12.0%), with a slightly higher incidence of stroke at 30 days for BAV patients (2.5% vs. 1.6%, *p* = 0.02), although not relevant at 1 year (3.4% vs. 3.1%, *p* = 0.16). Similarly, the BAV group had an acceptable device success rate (96.5%) and a marginally higher incidence of moderate or severe PVL compared with the tricuspid group (2.0% vs. 2.4%).

A similar analysis from the STS/ACC TVT Registry exploring the clinical and hemodynamic outcomes for patients with BAV stenosis undergoing TAVI with supra-annular self-expanding Evolut R valve or Evolut PRO valve (Medtronic) was conducted in 2020 by Forrest JK et al. [27]. A total of 929 patients with BAV anatomy treated with TAVI between 2015 and 2018 were matched with a tricuspid valve control group. No significant difference for all-cause mortality (30 days 2.6% vs. 1.7% and 1 year 10.4% vs. 12.1%) and stroke (30 days 3.4% vs. 2.7% and 1 year 3.9% vs. 4.4%) were observed. Comparable effective valve area was achieved between the two cohorts, with similar mean aortic gradient at 1 year (9.4 mmHg vs. 8.9 mmHg). However, a higher incidence of post-procedure moderate or severe aortic regurgitation (AR) was observed in patients with BAV (5.6% vs. 2.1%; *p* < 0.001) at 30 days, although this difference was mitigated in patients treated with the newer Evolut PRO valve. In this subgroup, AR rates were similar between BAV and tricuspid patients (2.2% bicuspid vs. 1.5% tricuspid; *p* = 0.71).

In 2017, Yoon et al. [28] performed a propensity score-matched analysis of 546 patients with bicuspid AS and tricuspid AS, all treated with TAVI from 2013 to 2017. In the bicuspid cohort, 30-day outcomes were similar, despite a significantly lower device success rate and a higher frequency of conversion to surgery compared with the tricuspid group. Interestingly, among patients receiving new-generation devices, no significant differences in procedural outcomes were noted between the bicuspid and tricuspid groups.

Montalto et al. [29], in a meta-analysis encompassing 19 studies and a total of 7071 matched subjects undergoing TAVI (3434 and 3637 with BAV and TAV, respectively), found no significant differences in 1 year all-cause of mortality and device success according to VARC-2 definition (RR: 0.91; 95% CI: 0.77–1.06; *p* = 0.23; and RR: 0.96; 95% CI: 0.91–1.01; *p* = 0.22). These results were consistent across device generation or valve type. Conversely, a significant higher rate of moderate to severe PVL, stroke or TIA, and annular rupture were observed in the BAV cohort, both in matched and unmatched populations.

Extended registries of the low-risk PARTNER 3 [31] and Evolut Low-Risk RCTs [35] confirmed comparable early outcomes compared with a matched cohort of patients with tricuspid aortic stenosis, without an available extended follow-up.

A propensity matched study from the “AD-HOC registry”, comparing clinical outcomes in patients with right-left (R-L) and right-non coronary (R-NC) raphe-type BAV treated with TAVI, found a three times higher incidence of permanent pacemaker implantation (PPI) in R-L compared with R-NC (16.1% vs. 6.7%; OR 0.37, CI 0.16–0.89), without any influences in major TAVI outcomes [30].

Recent data from the multicenter retrospective study by Li et al. [13], as mentioned before, show a better long-term prognosis (all-cause mortality) among patients with BAV-0 compared with BAV-1 (2-fold higher risk) and TAV (3-fold higher risk) after TAVI, especially when treated with SEVs. However, these results should be interpreted with caution considering the retrospective design of the study, the lack of complete follow-up of one-third of the population and the fact that BAV-0 patients were generally younger and healthier than the others [36].

Summary: Despite TAVI, outcomes in BAV and TAV stenosis are largely comparable in terms of mortality and procedural success, and bicuspid anatomy remains associated with higher risks of paravalvular leak, stroke, and annular complications, especially with older-generation devices.

### 5.3. SEV vs. BEV

A sub-analysis from the TAVI registry [28], comparing patients with bicuspid AS treated with early generation devices (the balloon expandable Sapien XT and the self-expandable supra-annular CoreValve) and newer generation device (Sapien3, Lotus, Evolut R) showed a more pronounced rate of procedural complications in the former group. Specifically, aortic root injuries were more frequent with Sapien XT implantation, whereas the choice of CoreValve prosthesis was associated with more episodes of second valve implantation and moderate or severe PVLs. Conversely, these differences were significantly reduced among patients implanted with newer generation devices.

An interesting analysis from the AD-HOC (Characteristics, Sizing, and Outcomes of Stenotic Raphe-Type Bicuspid Aortic Valves Treated With Transcatheter Device Implantation) registry [17,37] of 955 patients with Sievers type 1 BAV stenosis treated with TAVI, compared the outcomes after a propensity score–matching process between cohorts implanted with current-generation BEV versus SEV prostheses. Among the 301 matched paired patients, with a medium follow-up of 1.3 years, BEVs were associated with a lower risk of new permanent pacemaker implantation (OR: 0.42; 95% CI: 0.24–0.72; *p* = 0.002) and moderate or greater paravalvular regurgitation (OR: 0.16; 95% CI: 0.05–0.48; *p* = 0.001) at 30 days, compared with SEVs. However, the group of patients treated with BEVs showed a higher risk of severe patient-prosthesis mismatch (OR: 3.03; 95% CI 1.02–8.95; *p* = 0.045). No significant differences in procedural success or mortality at midterm follow-up were observed, although an overall higher rate of 30-day device success and early safety was noted for BEVs.

Interestingly, a parallel study by Zito et al. [32] from the same registry [17,37] found that in a cohort of Sievers type 1 BAV stenosis undergoing TAVI with current-generation THVs, the implantation of a self-expanding valve together with a larger virtual raphe ring perimeter and severe annular or left ventricular outflow tract calcification, was an independent predictor of moderate or severe PVL. At follow-up, patients with significant PVL had an increased risk of major adverse events (MAEs) compared with the mild PVL group.

Similar data were provided from the BEAT registry by Mangieri et al. [33], who compared patients with BAV (353 total, 77 matched pairs) undergoing TAVI using Evolut R/PRO or Sapien 3 valves. The study demonstrated a good success rate (85% VARC-2 device success) for both groups, but moderate or severe PVL was more common with SEV prosthesis, although they showed a better hemodynamic profile at discharge (lower mean gradient and higher effective orifice area). Conversely, a high number of annular ruptures with BEVs was observed, without differences in permanent pacemaker implantation. At 1-year follow-up, clinical outcomes were comparable between both groups.

The registry from Li et al. [13] suggested that among patients with BAV, there was a significant long-term survival advantage with SEVs compared with BEV prostheses, particularly in type 0 valves. However, given the several limitations described previously, these results should be interpreted with caution.

In this context, both operator experience and the use of high-quality pre-procedural imaging (including multidetector CT with standardized annular and supra-annular assessment) are critical for optimizing device selection and deployment strategy, thereby minimizing complications and improving outcomes.

Summary: Considering these data, both the current generation of SEVs and BEVs seem to be a viable option for TAVI in BAV, but with specific peculiarities. BEVs tend to offer lower rates of PVL, reduced need for permanent pacemaker implantation, but an increased risk of patient-prosthesis mismatch and annular injury. SEVs, on the other hand, provide better hemodynamic performance at the cost of a higher probability of significant PVL. Whether this translates to a better and longer durability of the valve it is not well-known. However, tailoring the choice of the device to the individual clinical and anatomical characteristics appears to be the only option for achieving good procedural and clinical outcomes.

## 6. Valve Durability After TAVI in BAV Patients

Anatomical challenges in BAV anatomy, like extensive calcium distribution and severe annular eccentricity, may impact device success and long-term valve durability after TAVI. This aspect is of paramount importance considering the fact that aortic valve deterioration typically happens earlier in BAV patients compared with TAV. The durability of transcatheter valves in patients with BAV remains a key concern, especially in younger populations. Current definitions of structural valve deterioration (SVD) have evolved to improve standardization. The 2021 EAPCI consensus document and the VARC-3 criteria define SVD based on objective hemodynamic changes, imaging evidence, and reintervention rates. These classifications allow comparison across studies, although inconsistencies in follow-up duration and echocardiographic thresholds still limit generalizability. In the bicuspid population, long-term data remain scarce, and further studies using harmonized definitions will be essential to assess prosthesis performance beyond 5–10 years.

### 6.1. Short-Term

Current data from the above-mentioned registries suggest acceptable outcomes in terms of device success and clinical outcomes at short/mid-term follow-up [17,27,32,33,37]. The 1-month data from the bicuspid arm of the LRT (Low Risk TAVR) trial [38], enrolling prospectively 61 symptomatic low-risk patients treated with TAVI (mainly balloon expandable devices), showed no mortality and disabling strokes. The reduction in mean aortic gradient and increase in aortic valve area were also maintained during the short-time follow-up.

Similar results regarding valve hemodynamic performances came from the BAVARD registry [15], where the 30-day post-TAVI mean gradient (9.4 ± 4.9 mmHg vs. 10.7 ± 4.9 mmHg; *p* = 0.15) and effective orifice area (2.1 ± 0.5 cm^2^ vs. 1.9 ± 0.6 cm^2^; *p* = 0.07) were comparable between BAV and TAV patients. High device success with a low rate of death or disabling stroke and good hemodynamic profile at 30 days were also confirmed by the study by Forrest et al. [35].

### 6.2. Long-Term

A recent single-center retrospective study published in 2024 by Li et al. [39] evaluated the long-term outcomes (mean follow-up of 5.1 years) of patients treated with TAVI in China comparing BAV versus TAV anatomies. Most of the implanted prostheses were first-generation self-expanding THVs (85–90%). No significant differences in terms of valve deterioration (BAV 11.8% versus TAV 11.0%) and bioprosthetic valve failure (BAV 1.8% versus TAV 4.8%) emerged during follow-up between the two groups, according to EAPCI/ESC/EACTs definitions [40]. However, moderate or greater PVLs were more common in the BAV group (6.5% versus 3.4%), with numerically fewer intra-valvular regurgitations (1.8% versus 4.8%) in the same population, without significant changes over follow-up. Data from the Italian The bicuSpid TAvi duraBILITY (STABILITY) registry [41] also suggested the good durability of TVH after TAVI in BAV patients, with low rates (4%) of hemodynamic valve dysfunction (HVD), according to the valve aortic research consortium (VARC)-3 update definitions [42], despite a slight increase in the mean transprosthetic gradient over 4-year follow-up.

Summary: Current data regarding the long-term performance and durability of transcatheter implanted bioprosthesis in BAV patients are encouraging, despite being limited. However, more studies with long-term follow-up are required to confirm this trend.

## 7. Limitation of Current Evidence

Despite the growing evidence supporting the use of TAVI in BAV disease, most available data are derived from observational registries, retrospective cohort studies, and post hoc subgroup analyses of larger trials not primarily designed for this population. These studies often suffer from selection bias, as patients selected for TAVI in the setting of BAV tend to be highly selected and treated in experienced centers. Moreover, the retrospective nature of many analyses introduces confounding factors that cannot be fully adjusted for, particularly regarding anatomical complexity and procedural decision-making. Device heterogeneity is another major limitation, as different studies include multiple generations of transcatheter valves with varying designs, delivery systems, and sizing strategies, making comparison across datasets challenging. Incomplete and inconsistent follow-up, especially beyond 1–2 years, further limits the ability to draw robust conclusions on mid- and long-term outcomes such as valve durability, reintervention rates, and survival. Finally, echocardiographic evaluation and endpoint definitions (e.g., structural valve deterioration, paravalvular leak, stroke) may differ across studies, limiting standardization. These limitations underscore the need for prospective, randomized studies with dedicated BAV cohorts and standardized follow-up protocols to provide more definitive evidence for this complex patient group.

## 8. Conclusions

Bicuspid aortic stenosis remains a challenging scenario for interventional cardiologists. TAVI has emerged as a feasible option for BAV patients, especially if elderly with a high-risk profile for surgery and with suitable anatomy for percutaneous approach. However, with the current spread of TAVI to younger and low-risk population, the number of patients with BAV being considered for a percutaneous approach is expected to increase.

Although the majority of data was derived from non-randomized retrospective registries, the available evidence is encouraging, suggesting comparable device success and short- to mid-term outcomes for TAVI in patients with BAV. Nevertheless, procedural complications, including PVL and conduction abnormalities, remain more frequent compared with tricuspid anatomies, although mostly limited to first-generation devices. Adequate pre-procedural imaging, device selection, and case-by-case per-procedural planning are crucial to improve the outcomes after TAVI. Still, further randomized, large-scale, long-term studies are necessary to confirm the feasibility and durability of TAVI across the wide spectrum of BAV population.

## Figures and Tables

**Table 1:** Pre-procedural sizing methods for TAVI in bicuspid anatomy.

Method	Measurement Plane/Rule	Core Concept and Workflow	Main Advantages	Main Drawbacks
**BAVARD**	Inter-commissural distance (ICD) 4 mm above annulus; Classify tapered/tubular/flared configurations	If root is tapered, sizing follows ICD (smaller value); in the other cases (88%), annulus guides sizing	Avoids oversizing in tapered roots; validated in both SE and BE THV; suitable for type 0 BAV	ICD measurement at a fixed level (4 mm), independently from interindividual variability; based on mono-dimensional parameter (diameter vs. perimeter/area)
**LIRA**	Maximal raphe protrusion plane (~10 mm supra-annular), choose smaller of LIRA vs. VBR perimeter	Compare supra-annular perimeter (LIRA) with VBR; THV size = smaller perimeter-derived Ø	Accurate THV waist prediction; high device success; low PVL	Applicable only to raphe-type BAV and for SE THVs; limited cohort size
**CASPER**	Start from annulus Ø, subtract 0–2.5 mm based on: calcium >300 mm^3^, raphe length >50%, calcium on raphe	Algorithmic downsizing of annulus Ø according to calcium burden and distribution on raphe	Personalized sizing; low PVL/embolization in pilot studies	Requires calcium-scoring software; not validated for BE THVs and applicable only to raphe-type BAV
**CIRCLE**	Overlay device-sized circles every 3 mm above annulus; first full-contact circle defines size	Visual sizing tool (balloon-expandable THVs) and assessment of coronary/LVOT clearance	Fast, intuitive; highlights anatomic conflicts; suitable for type 0 BAV	Empirical; not validated for self-expanding THVs

Abbreviations: BAV = bicuspid aortic valve; BAVARD = Bicuspid Aortic Valve Anatomy and Relationship With Devices; BE = balloon-expandable; CASPER = Calcium Algorithm Sizing for bicusPid Evaluation with Raphe; ICD = inter-commissural distance; LIRA = Level of Implantation at the RAphe; LVOT = left ventricular outflow tract; SE = self-expandable; PVL = paravalvular leak; THV = transcatheter heart valve; VBR = virtual basal ring.

**Table 2:** Practical sizing strategies and THV selection in bicuspid aortic valve anatomy.

Features/Scenario	Sizing and Device Selection Tips
Sievers Type-0 BAV (no raphe)	Symmetric oval annulus; standard annular sizing with minimal oversizing (≈0–5%).
Sievers Type-1 BAV (one raphe)	Asymmetric anatomy with calcified raphe; use supra-annular (LIRA) measurements for sizing. Consider self-expanding valve to achieve larger EOA. Avoid excessive oversizing (>5%).
Bulky commissural calcification	Very high-risk for underexpansion. Favor downsizing (e.g., 0–5% oversize) to prevent rupture.
Elliptical (oval) annulus	High ellipticity; lean toward the smaller size to avoid distortion. Supra-annular THV can help accommodate shape.
Supra-annular valve (e.g., Evolut/Accurate)	Provides larger EOA and lower gradients. Good choice for small annuli, severe gradients or when preserving coronary access. Anchor depth should be planned at raphe level in BAV.
Intra-annular valve (balloon-expandable)	Offers precise deployment but requires caution in heavy calcification (risk of rupture). Use minimal oversizing and careful valve positioning.

**Table 3:** Key evidence summary for TAVI in bicuspid anatomy.

	Study (Year)	Study Design	N (Patients)	BAV Type (Sievers)	THV(s)	Follow-Up	Key Results
**TAVI vs. SAVR**	**Elbadawi et al., 2019 [22]**	Propensity-matched registry	975 pairs	NR	Mixed early-gen BEV and SEV	In-hospital	Mortality 3.1% vs. 3.1%. ↑ conduction disorders (14.9% vs. 6.2%) and pacemaker (13.8% vs. 4.6%) with TAVI
	**Mentias et al., 2020 [23]**	Propensity-matched registry	699 pairs	NR	Mixed BEV and SEV	30 day and 1 y	No Δ mortality/stroke and HF hospitalization at 1 y; ↑ pacemaker (12.2% vs. 7.6%) with TAVI
	**Majmundar et al., 2022 [24]**	Propensity-matched registry	1393 pairs	NR	Current-gen BEV and SEV	In-hospital, 30-day, 6 months	↓ in-hospital mortality (0.7% vs. 1.8%) in TAVI. Similar 30-d and 6-mo MACE;
	**NOTION-2 Trial, 2024 [25]**	Randomized clinical trial (low-risk)	100 BAV (49 TAVI vs. 51 SAVR)	Predominantly Type 1	Mixed new-gen BEV and SEV	1 y	Trend to ↑ composite death/stroke/rehospitalization (14.3% vs. 3.9%); ↑ disabling stroke, ↑ moderate/severe PVL but less PPM with TAVI;
**TAVI** **(BAV vs. TAV)**	**NOTION-2 2024 (sub-analysis) [25]**	Randomized clinical trial (subgroup)	100 BAV vs. ~270 TAV	Predominantly Type 1	Mixed BEV and SEV	1 y	≥Moderate PVL 9.1% vs. 3.1%; Pacemaker similar; Composite endpoint numerically higher in BAV
	**Makkar et al., 2021 [26]**	Propensity-matched registry	3168 pairs	Mixed Type 0/1 (~80% Type 1)	BEV (Sapien 3)	30 day and 1 y	Similar 30-d and 1-y mortality; ↑ stroke (2.5% vs. 1.6%) at 30-d for BAV; ↑ moderate/severe PVL 2.4% vs. 2.0% for BAV
	**Forrest et al., 2020 [27]**	Propensity-matched registry	929 pairs	Predominantly Type 1	SEV (Evolut R/PRO)	30 day and 1 y	Mortality/stroke similar; ↑ moderate/severe AR 5.6% vs. 2.1% at 30 d (mitigated with PRO)
	**Yoon et al., 2017 [28]**	Propensity-matched multicenter	546 pairs	73% Type 1; 27% Type 0	Early and new-gen BEV and SEV	30 day	Similar 30-d outcomes; Lower device success and ↑ conversion to surgery in BAV (gap closed with new-gen THVs)
	**Montalto et al., 2021 [29]**	Systematic review and meta-analysis	7071 (3434 BAV, 3637 TAV)	Mixed (0, 1, 2)	Mixed	1 y	No differences of 1-y mortality/device success; ↑ PVL, stroke/TIA and annular rupture in BAV
	**Vicerè et al., 2025 [30]**	Propensity-matched registry	382 pairs among raphe type BAV (251 R-L, 131 R-NC BAV)	Type 1 only	Mixed	In-hospital, 30-day, mid-term	No differences of 30-day VARC-3 technical/device success; Comparable short- and mid-term outcomes. ↑ PM implantation in R-L phenotype.
	**Forrest et al., 2020 (Low-Risk BAV) [27]**	Prospective single-arm	150 patients (Single-arm (no control)	Type 1 ≈91%	SEV (Evolut/Evolut PRO)	30 day	Death/disabling stroke 1.3%; device success 95.3%; pacemaker 15.1%; no >mild PVL
	**Williams et al., 2022 (PARTNER 3 BAV) [31]**	Prospective registry, matched	169 BAV (148 matched)	Type 1	BAV vs. matched TAV (BE THV)	1 y	Primary endpoint 10.9% vs. 10.2% (ns); death 0.7% vs. 1.4%; stroke 2.1% vs. 2.0%; rehosp 9.6% vs. 9.5%
	**Li et al., 2025 [13]**	Multinational retrospective cohort	2553 (134 BAV-0; 305 BAV-1; 2114 TAV)	Type 0 versus Type 1 and TAV	Mixed BEV and SEV	Median ~3.2 y (5-year endpoints)	BAV-0 ↓ 5-year all-cause mortality compared with BAV-1 (adjusted HR ~2.4) and TAV (adjusted HR ~3.0)
**SEV vs. BEV**	**Yoon et al., 2017 (device sub-analysis) [28]**	Registry sub-analysis	546	73% Type 1; 27% Type 0	Early-gen Sapien XT (BEV) vs. CoreValve (SEV)	30 d	Early BEV: ↑ root injury; Early SEV: ↑ second valve and PVL; gaps reduced with new-gen devices
	**Buono et al.,** **2024 (AD-HOC registry) [17]**	Propensity-matched registry	301 pairs	Type 1 only	BEV (Sapien 3) vs. SEV (Evolut R/PRO)	Median 1.3 y	BEV: ↓ pacemaker (OR 0.42) and PVL (OR 0.16) but ↑ severe PPM mismatch (OR 3.03); Similar mortality
	**Zito et al.,** **2024 (AD-HOC registry) [32]**	Registry analysis	955	Type 1 only	Predominantly current-gen THVs	1 y	SEV + large raphe and heavy calcification → independent predictor of ≥moderate PVL; subgroup with significant PVL >> ↑ MAE
	**Mangieri et al., 2020 (The BEAT registry) [33]**	Matched registry	77 pairs	Predominantly Type 1	SEV (Evolut R/PRO) vs. BEV (Sapien 3)	1 y	Device success 85% both; ↑ PVL with SEV; ↑ annular rupture with BEV; SEV better hemodynamics; outcomes similar
	**Li et al., 2025 [13]**	Multinational retrospective cohort	2553 (134 BAV-0; 305 BAV-1; 2114 TAV)	Type 0 versus Type 1 and TAV	Mixed BEV and SEV	Median ~3.2 y (5-year endpoints)	BEVs in BAV ↑ mortality vs. SEVs (41.7% vs. 23.6%, HR ~1.63)

Abbreviations: AR = aortic regurgitation; BAV = bicuspid aortic valve; BEV = balloon-expandable valve; Δ (delta) = difference/change; d/mo/y = days/months/years (follow-up units); MAE = major adverse events; MACE = major adverse cardiovascular events; NR = not reported; ns = not significant; OR = odds ratio; PPM mismatch = patient–prosthesis mismatch; PVL = paravalvular leak; R-L = right-left raphe-type BAV; R-NC = right-non coronary (R-NC) raphe-type BAV; SAVR = surgical aortic valve replacement; SEV = self-expanding valve; TAV = tricuspid aortic valve; TAVI = transcatheter aortic valve implantation; THV = transcatheter heart valve.

## Data Availability

No new data were created or analyzed in this work.
